# Developing a trigger tool to monitor adverse events during haemodialysis in children: a pilot project

**DOI:** 10.1007/s00467-022-05673-4

**Published:** 2022-08-01

**Authors:** Ramnath Balasubramanian, Rachel Folwell, Arran Wheatley, Heidi Ramsey, Carmen Barton, Christopher J. D. Reid, Manish D. Sinha

**Affiliations:** 1 Department of Paediatric Nephrology, Evelina London Children’s Hospital, Guys & St Thomas’ NHS Foundation Trust, 3rd Floor Beckett House, Westminster Bridge Road, London, SE1 7EH UK; 2 Kings College London, London, UK

**Keywords:** Haemodialysis, Trigger tool, Adverse events

## Abstract

**Abstract:**

**Background:**

We developed a paediatric haemodialysis trigger tool (pHTT) for application per haemodialysis (HD) session in children receiving intermittent in-centre HD and systematically monitored adverse events.

**Methods:**

Single-centre quality improvement study performed over two 8-week cycles. Data collected prospectively using a ‘per-dialysis session’ pHTT tool including 54 triggers across six domains, adapted from a recently described haemodialysis trigger tool (HTT) for adults. Each trigger was evaluated for level of harm following assessment by two authors. Following a period of training, HD nurses completed the HTT at the end of each dialysis session.

**Results:**

There were 241 triggers over 182 dialysis sessions, with 139 triggers in 91 HD sessions for 15 children, age range 28–205 months, over an 8-week period (first cycle) and 102 triggers in 91 HD sessions for 13 children, age range 28–205 months, over a further 8-week period (second cycle). After interventions informed by the pHTT, the harm rate per session was significantly reduced from 1.03 (94/91) to 0.32 (29/91), *P* < 0.001. There was a significant difference between the distribution of triggers by harm category (*P* < 0.001) and between the proportion of triggers across the various domains of the pHTT (*P* = 0.004) between the two cycles. No triggers were evaluated as causing permanent harm.

**Conclusions:**

This pilot study demonstrates potential benefits of a bedside tool to monitor adverse events during haemodialysis in children. Thus, following interventions informed by the pHTT, the harm rate per session was significantly reduced. Under standard patient safety systems, the vast majority of triggers identified by the pHTT would remain unreported and perhaps lead to missed opportunities to improve patient safety. We propose the use of a paediatric HTT as part of standard care by centres providing HD to children in the future.

**Graphical Abstract:**

A higher resolution version of the Graphical abstract is available as Supplementary information

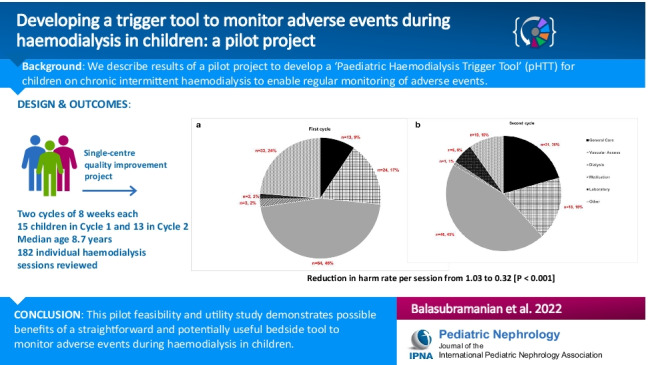

**Supplementary Information:**

The online version contains supplementary material available at 10.1007/s00467-022-05673-4.

## Introduction

Haemodialysis is a life-sustaining procedure in the management of children with kidney failure. However, it can be associated with multiple complications including intradialytic hypotension, problems with vascular access, infection, and adverse cardiovascular outcomes [1]. Often the reporting of these complications remains limited to only serious adverse events or critical incidents only. Potential harm events are likely to go unreported as they may be considered minor, inconsequential, or unrelated to a recognised adverse event at the time of reporting. Furthermore, without systematic reporting of all adverse events, risk patterns may not be recognised. The ‘Global Trigger Tool’ introduced by the Institute for Healthcare Improvement has been widely used in adult inpatient healthcare settings to monitor and reduce harm [2]. Potential adverse events are identified by reviewing notes respectively for ‘triggers’ and their rates measured over time. This tool was shown to have utility in an inpatient paediatric population with 25.8% of patients experiencing at least one adverse event during hospitalisation [3]. Trigger tools in children have subsequently been used in other in-patient hospital settings including paediatric and neonatal intensive care [4–6].

We hypothesise that in patients receiving long-term in-centre intermittent haemodialysis, systematic case note review could identify trends in harm events, guide practice development, and enhance patient safety. In this report, we describe results of a pilot quality improvement project to develop a ‘Paediatric Haemodialysis Trigger Tool’ (pHTT) for children on chronic intermittent HD to enable regular monitoring of adverse events. Our objectives were to (i) develop a paediatric HD trigger tool (pHTT) for application per haemodialysis session in children receiving intermittent in-centre HD; and (ii) systematically monitor adverse events during HD sessions using the pHTT and determine the frequency and severity of harm.

## Methods

Our paediatric nephrology centre provides comprehensive acute and chronic dialysis, apheresis and kidney transplantation services to Southeast England serving a population of over 1.7 million children and adolescents. A core team of haemodialysis specialist nurses provides dialysis care. We also have a small number of children’s kidney nurses, less experienced in dialysis treatments, working on the unit regularly. There is usually one experienced dialysis nurse for every two patients receiving haemodialysis and one nurse per patient for complex therapies, clinically unstable, or young children.

### Development of trigger tool

An HD Trigger Tool (HTT) to detect and monitor harm events specific to HD has been developed for adults receiving in-centre HD in Derby, UK [7]. The adult HTT was discussed extensively at our departmental dialysis staff meeting, with input from dialysis nurse specialists. The adult HD trigger tool was adapted for a paediatric HD population and also includes additional local safety practices. We produced a ‘per-dialysis session’ pHTT, referred to as the ‘The Evelina London pHTT’, that includes 54 trigger events across six domains: general care, vascular access, dialysis, medication, laboratory tests, and other (Table 1). Individual triggers in each domain were adapted to the paediatric population (for example, post-HD weight targets; ultrafiltration rate). We also incorporated local operating procedures into the HTT — for example, our standard practice to review medications and blood results with the parent or caregiver weekly, and our daily ‘safety huddle’ of all dialysis staff.

**Table 1:** Demographic characteristics of children on chronic intermittent haemodialysis included in the two cycles of the pilot study

	Cycle 1	Cycle 2
Number of children	15	13
Age median (IQR), years	9.7 (4.7, 11.6)	7.7 (5.0, 12.7)
Duration on haemodialysis median (IQR), years	1.0 (0.4, 2.2)	1.7 (0.8, 3.3)
Sex		
Male, *n* (%)	9 (60%)	7 (54%)
Female, *n* (%)	6 (40%)	6 (46%)
Primary renal disease		
CAKUT, *n* (%)	5 (33%)	5 (38%)
Glomerular, *n* (%)	6 (40%)	5 (38%)
Other, *n* (%)	4 (27%)	3 (24%)
Number of HD sessions reviewed	91	91

*CAKUT*, congenital anomaly of kidney and urinary tract; *HD*, haemodialysis

The ‘safety huddle’ is a mid-morning update between all nurses on the unit, ideally once all the patients are on their machines and before any staff go on breaks. Typically, this is a 15-min meeting (‘huddle’) to discuss the patients’ weight, access, investigations completed or due, social concerns, and time planned for the session. This provides an opportunity for the nurse-in-charge to review the dialysis session plan, ensure medications are given and blood results are checked, and any concerns are escalated appropriately. It also informs the rest of the nurses about each patient’s plan for the session so they can work as a team/cover breaks.

**Fig. 1:**
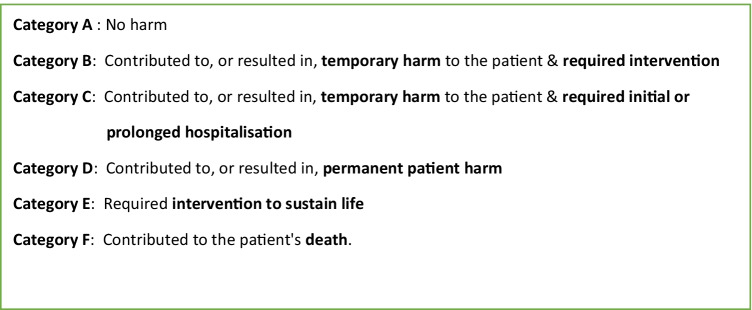
The categorisation of each trigger for level of physical harm using the ‘per dialysis session’ paediatric Haemodialysis Trigger Tool (pHTT) adapted from the Derby HD Trigger Tool (HTT) [7]

**Fig. 2:**
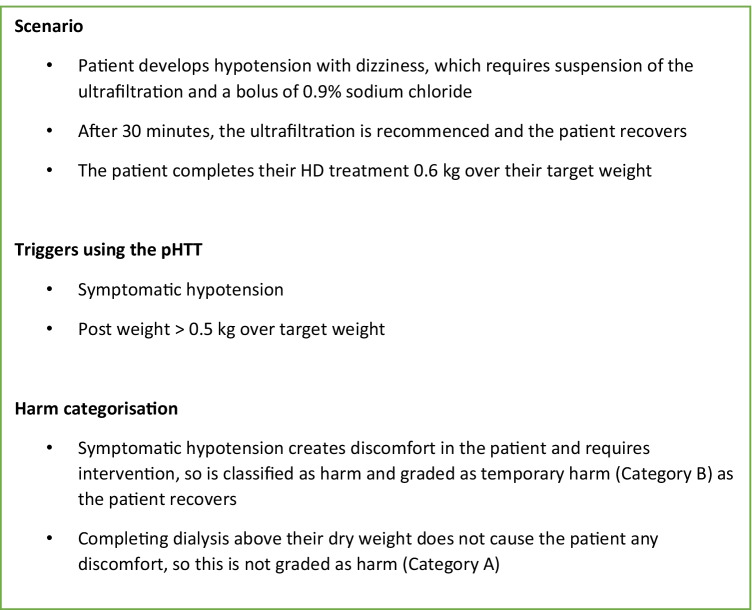
Example of the application of the ‘per dialysis session’ paediatric Haemodialysis Trigger Tool (pHTT) during a typical in-centre haemodialysis session. This example is of one single haemodialysis session, showing how the harm rating was arrived at for that session. The level of harm was assigned based on the outcome at the end of that specific session. It is though logical to assume that a series of such “no harm events” are likely to result in a “harm event” in the future

Each trigger was evaluated for level of physical harm and categorised as shown in Fig. 1. We defined harm as anything caused by a medical intervention that caused discomfort or symptoms for the patient or required intervention to correct. We categorised harm as (i) no harm (category A); (ii) temporary harm requiring intervention (categories B and C); or (iii) permanent harm (categories D, E, and F). Preliminary use of the pHTT tool was conducted by two dialysis nurses who confirmed ease of use, minimal time commitment, and no significant addition to their workload. All HD nurses at our centre were trained to complete the pHTT, and completed the pHTT at the end of each HD session in < 5 min per patient. Allocation to harm categories was undertaken independently by two authors and any differences between them discussed, and then a single category was agreed upon. An example of how different harm categories were allocated to a trigger event is illustrated in Fig. 2.

### Trigger tool pilot cycles

Prospective data collection for all children receiving in-centre HD was performed over an initial 8-week period. A second 8-week cycle of using the pHTT was repeated after a 3-month interval. The results of both cycles are reported here. The results of the pHTT were reviewed by a member of the medical team on a weekly basis.

### Data collection

Data are displayed as total number (and percentage), mean with 95% confidence interval, or median and interquartile range. We calculated the ‘trigger rate per session’ over each cycle and defined it as the number of triggers divided by the number of HD sessions. We calculated the ‘harm rate per session’ over each cycle and defined it as the number of triggers resulting in harm (temporary harm requiring intervention; or permanent harm (categories B–E)) divided by the number of HD sessions over each cycle. Statistical significance was considered if *P* ≤ 0.05.

## Results

### First cycle

Over the first 8-week cycle, the pHTT was completed for 91 HD sessions for 15 children (Table 1). One hundred and thirty-nine potentially harmful trigger events were identified (Table 2). Forty-five of these events (32.4%) were categorised as a ‘no harm’ event (category A); 92 (66.2%) were categorised as ‘temporary harm requiring intervention’ (category B); and 2 (1.4%) were categorised as ‘temporary harm requiring initial or prolonged hospitalization’ (category C). No events resulted in permanent harm (categories D, E, and F). Over the first cycle, the top 3 triggers were ‘failure to have nursing safety huddle during HD session’ 16.5% (23/139) in the other domain category; followed by ‘required UF rate > 5% or need for IUF’ 10.8% (15/139) in the dialysis domain; and ‘need for TPA’ 9.4% (13/139) in the vascular access domain.

**Table 2:** Distribution of 241 triggers over 182 dialysis sessions using the ‘per dialysis session’ Evelina London paediatric Haemodialysis trigger tool (pHTT) over two 8-week periods including *n* = 15 (first cycle) and *n* = 13 (second cycle) patients receiving chronic in-centre haemodialysis

General care	Triggers	Cycle 1	Category A	CategoryB	Category C	Cycle 2	Category A	Category B	Category C
G1	Vomiting	2	2			3		3	
G2	Fever > 38 C	2		1	1				
G3	Symptomatic hypotension	5		5		16		16	
G4	Loss of consciousness								
G5	Unplanned admission post-HD					1			1
G6	Chest pain	2		2					
G7	Cardiac arrest								
G8	Cardiac arrhythmia								
G9	Emergency medical assessment	1		1		1		1	
G10	Blood transfusion								
G11	Oxygen therapy	1			1				
G12	Fall pre/post-HD								
G13	Seizures								
	Total	13	2	9	2	21	0	20	1
Vascular access	Triggers	Cycle 1	Category A	Category B	Category C	Cycle 2	Category A	Category B	Category C
V1	More than 1 attempt to place needle	1		1		2		2	
V2	Single needle dialysis/lines reversed	8		8		7	7		
V3	Haematoma								
V4	Infected fistula								
V5	Exit site infection					1		1	
V6	Thrombosed/occluded catheter								
V7	Prolonged bleeding post-HD								
V8	Venous needle dislodgement	1		1					
V9	Need for TPA	13		13		8	8		
V10	Fistula not buzzing	1		1					
	Total	24		24		18	15	3	0
Dialysis	Triggers	Cycle 1	Category A	Category B	Category C	Cycle 2	Category A	Category B	Category C
D1	Failure to complete >90% of prescribed time	1		1		4	1	3	
D2	BFR<75% of normal	3		3		6	6		
D3	Loss of circuit	1		1					
D4	Machine failure (replacement)	8		8					
D5	Water treatment failure								
D6	Delayed start of treatment <30 min	6		6		4	4		
D7	Delayed start of treatment >30min	2		2		1	1		
D8	Post-HD weight >0.5kg above target	8		8		9	9		
D9	Post HD weight >0.5kg below target					3	3		
D10	Required UF rate >5% or need for IUF	15		15		11	10	1	
D11	Need for extra SCUF session	4		4		5	5		
D12	Dialysis prescription error	2		2					
D13	Missed treatment								
D14	High VP/TMP — causing high/extra UFR	2		2					
D15	Comes in below dry weight	12	7	5		3	3		
	Total	64	7	57	0	46	42	4	0
Laboratory	Triggers	Cycle 1	Category A	Category B	CategoryC	Cycle 2	Category A	Category B	Category C
L1	Hypoglycemia								
L2	Hypokalemia <3.0 mmol/l preHD								
L3	Hyperkalemia > 6.5mmol/l preHD					3	3		
L4	Hypocalcemia <1.9 mmol/l	1		1		1	1		
L5	Hypercalcemia >2.8 mmol/l	1		1					
L6	Positive blood culture					1		1	
L7	Haemoglobin < 80 g/l					1	1		
L8	Haemoglobin >140g/l								
	Total	2	0	2	0	6	5	1	0
Medication	Triggers	Cycle 1	Category A	Category B	Category C	Cycle 2	Category A	Category B	Category C
M1	Oral nifedipine dose given								
M2	IV antibiotics — HD related								
M3	Medication review with parent not done	3	3			1	1		
M4	Prescribed medication review not performed								
	Total	3	3	0	0	1	1	0	0
Other	Triggers	Cycle 1	Category A	Category B	Category C	Cycle 2	Category A	Category B	Category C
OH1	Blood result review with parent not done	3	3			1	1		
OH2	Delayed blood results from lab	7	7						
OH3	No safety huddle	23	23			9	9		
	Total	33	33	0	0	10	10	0	0
	All triggers	**139**	**45**	**92**	**2**	**102**	**73**	**28**	**1**

*HD*, haemodialysis; *TPA*, tissue plasminogen activator; *BFR*, blood flow rate; *UF*, ultrafiltration; *UFR*, ultrafiltration rate; *IUf*, increased ultrafiltration; *SCUf*, slow continuous ultrafiltration; *VP*, venous pressure; *TMP*, transmembrane potential; *IV*, intravenous

Following this cycle, the results were presented to the HD team and the following interventions were made to reduce triggers and subsequent harm events, including (i) regular multi-disciplinary dialysis education days; (ii) regular re-evaluation of estimated target weights; (iii) adherence to safety huddles; and (iv) discussion with laboratory, hospital information technology team and biomedical teams to reduce delays in obtaining laboratory results, and reduce HD machine failures.

### Second cycle

The second cycle of the pHTT was performed after a 3-month interval. The pHTT was completed for 91 HD sessions for 13 children. Ten of 15 children who were included in the first cycle were on dialysis at the time of the second cycle. The remaining 5 patients did not receive HD during the second cycle for various reasons (received a transplant, changed dialysis modality, or transferred to another HD unit). Three new patients were included in the second cycle. One hundred and two potentially harmful trigger events were identified (Table 2). Seventy-three of these events (71.5%) were categorised as ‘no harm’ events (category A), and 28 (27.4%) were categorised as ‘temporary harm requiring intervention’ (category B). One event (1%) was categorised as ‘temporary harm requiring initial or prolonged hospitalization’ (category C).

During this second cycle, the top 3 triggers included ‘need for additional fluid removal’ in the dialysis domain and ‘symptomatic hypotension’ in the general care domain at 15.7% each (16/102 each); followed by ‘post HD weight more than 5% above target’ 8.8% (9/102) in the dialysis domain.

**Fig. 3:**
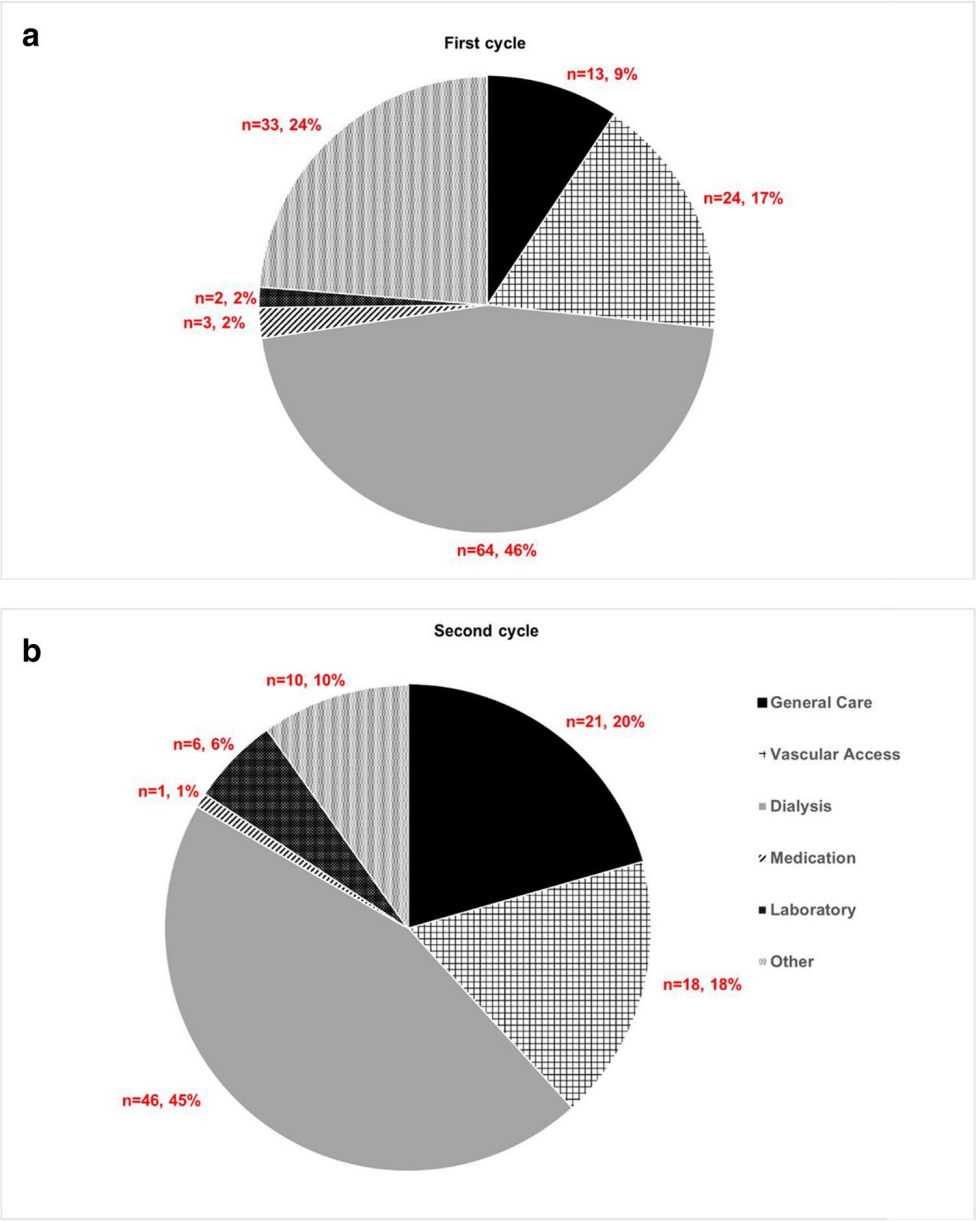
Frequency of observed triggers across the six domains of the ‘per dialysis session’ Evelina London paediatric Haemodialysis Trigger Tool (pHTT) over two 8-week periods including (**a**) *n* = 15 (first cycle) and (**b**) *n* = 13 (second cycle) patients receiving chronic in-centre haemodialysis

The mean trigger rate per session during the first cycle was 1.53, with 95% confidence interval (1.32–1.74). In the second cycle, the mean trigger rate per session had reduced to 1.12, with 95% confidence interval (0.83–1.41), *P* = 0.001. The harm rate per session was significantly reduced from 1.03 (94/91) to 0.32 (29/91), *P* < 0.001. There was a significant difference between the proportion of triggers across the various domains of the pHTT over the two cycles (*P* = 0.004) (Fig. 3).

## Discussion

To our knowledge, this is the first study to evaluate the feasibility and clinical utility of a specific paediatric haemodialysis trigger tool. Using the pHTT to guide interventions, we observed a reduction both in the trigger and harm rates per session. This correlates with improved safety and care of children receiving in-centre intermittent haemodialysis.

Over the course of this pilot study, there were only 3 events which resulted in hospitalisation (2 and 1 during the first and second cycles, respectively) representing only 1.2% (3/241) of all reported trigger events during the study period. If the standard patient safety reporting mechanisms were followed, only these ‘severe’ adverse events would have been reported. All other triggers resulting in an intervention that may have caused temporary harm would have gone unreported. These data highlight the ability of a paediatric dialysis-specific trigger tool to capture adverse events and underscore its clinical utility.

We observed changes in the distribution of the trigger events across harm categories. This may be related to differences between patients over the two cycles or as a result of ongoing interventions. Our findings are similar to an adult HTT study, which also reported a significant reduction in the severity and harm rate of triggers [7]. However, it is important to highlight that the trigger rate per HD session observed in this paediatric HD population is higher than the study of an adult HD cohort, in which the trigger rate per session was 0.71. These data highlight the relative complexities in delivering HD to children when compared to adults. Differences between paediatric and adult HD populations are also reflected in the differences between observed triggers. While the need for additional fluid removal was the most frequent trigger in children, symptomatic hypotension was most commonly observed in adults [7]. The need for additional fluid removal reflects non-compliance with fluid and dietary restrictions in some children in this study. Given the association of volume overload with increased morbidity and mortality, this observation emphasises the need for close clinical monitoring and repeated assessment and probing of the estimated dry weight [8, 9]. Arriving for the HD session below target weight was also common. This likely relates to the significant numbers of children on HD who remain polyuric as CAKUT is a common cause of kidney failure [10].

The pHTT can also be used as a tool to measure quality of care in the HD unit. Following the first cycle, we addressed logistical issues including regular safety huddles, ensuring prompt turnaround of laboratory results, and machine failures. These interventions were reflected in the lower trigger and harm rates in the second cycle. However, the frequency of other trigger events increased, reflecting challenges in achieving target weights and adequate ultra-filtration. Overall, the value of the trigger tool is reflected in the awareness of ‘low level’ harm or near misses which collectively demonstrate trends and areas for improvement.

The limitations to this pilot study are inherent to those seen in small study numbers and data from a single centre. We accept the fact that small patient numbers in our study are likely to impact our observations. For example, a single patient contributed to the increase in the trigger for symptomatic hypotension when comparing the first versus the second cycle [3.6% versus 15.6%]. The patient population was different between the two cycles, although the number of dialysis sessions comparable. Although a limitation of the study, change in patient population is observed in all paediatric HD centres. Despite these limitations, both trigger rate per session and harm rates were significantly reduced over the second study period. These findings are similar to those reported for the adult HTT, which also identified a long-term reduction in harm and trigger incidence [7]. This reduction was attributed to increasing staff awareness and vigilance of the triggers that might cause harm to patients, through feedback to staff of the results. We anticipate a similar trend in our unit and it is a focus of ongoing work. We acknowledge missing medicines, incorrect doses, and discrepancies between what is prescribed and what is actually given to the patient as potential triggers. We plan to add these as additional triggers in further iterations of the trigger tool. In addition, including parent views to develop additional triggers would also be an important aspect not included in the pHTT in its current version. Our findings will need to be tested in larger studies and adapted for use in other centres to measure clinical impact and potential financial benefits as a result of reduction of interventions in the dialysis unit or prevention of hospitalisations. Nevertheless, these observations are novel and outline a systematic method to monitor overall clinical care across several domains.

## Conclusion

Implementation of this pHTT provides evidence of the risks inherent to paediatric HD and the value of regular monitoring of adverse events. This pilot study demonstrates possible benefits of a potentially useful bedside tool to monitor adverse events during haemodialysis in children. Thus, following interventions informed by the pHTT, the harm rate per session was significantly reduced. We propose that a pHTT be included as part of standard care by centres providing HD to children. We anticipate individual centres would modify this pHTT prior to implementation so that it is aligned with local operating policies and procedures. We would recommend that centres consider full adoption of the current version of the pHTT, or the use of consistent core parameters to monitor with some additional site-specific factors. We recommend performing the pHTT regularly as a risk assessment tool and as a measure of patient safety but further studies are needed to judge the optimal frequency.

## Supplementary Information

Graphical Abstract (PPTX 253 kb)

## Declarations

The authors confirm that data collected was part of the provision of routine clinical care and as part of this quality improvement project analysed retrospectively following delivery of clinical care. No consent from patients was indicated and ethical approval was not required.
