# Supplementary material for: Developing a trigger tool to monitor adverse events during haemodialysis in children: a pilot project
Source: Pediatr Nephrol. 2022 Aug 1;38(4):1233–40. doi: 10.1007/s00467-022-05673-4 (PMC9925574; doi:10.1007/s00467-022-05673-4)
Supplement: Supplementary file 1 — (PPTX 253 kb) [file 467_2022_5673_MOESM1_ESM.pptx]

## Slide 1
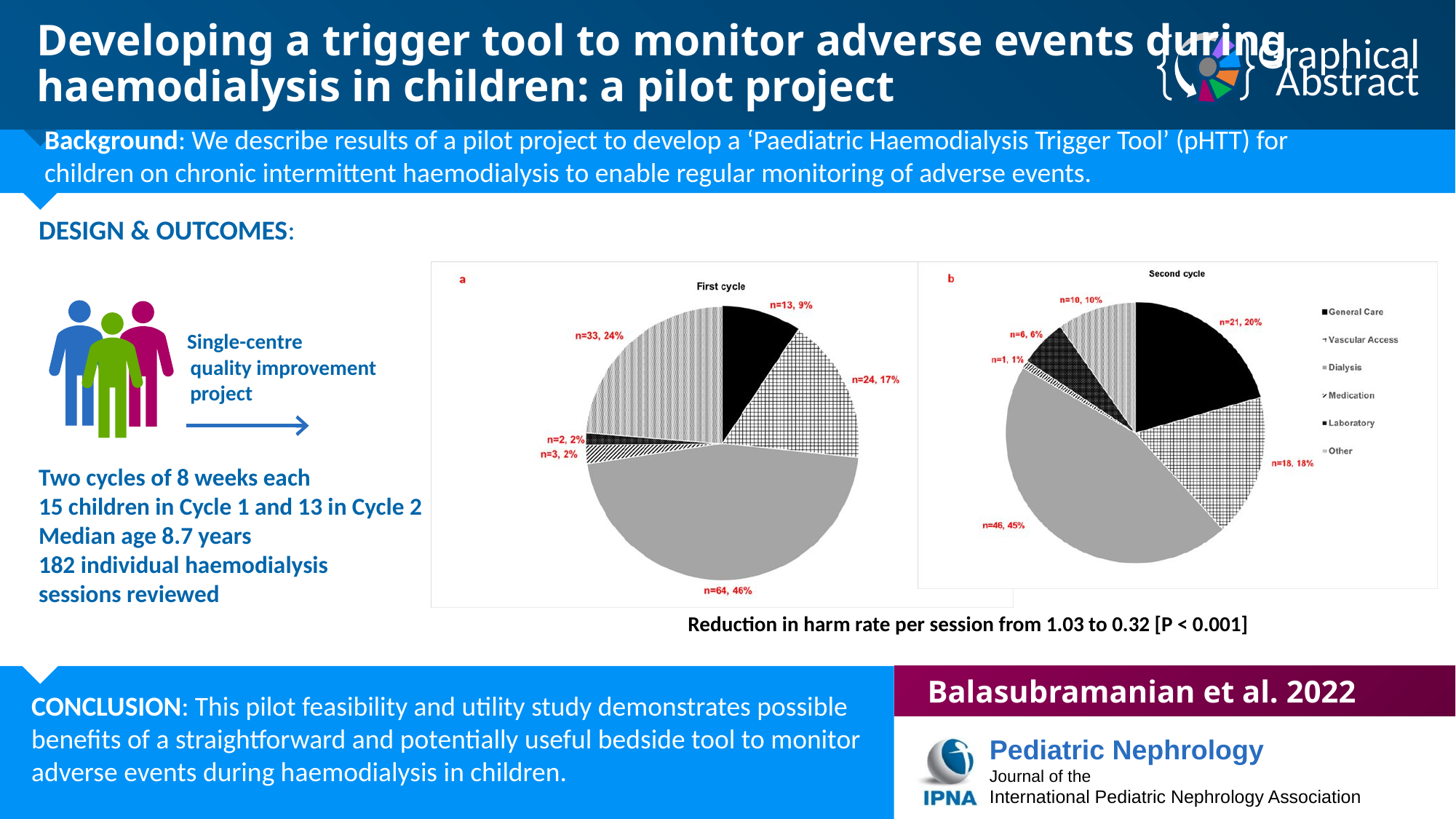

Developing a trigger tool to monitor adverse events during
haemodialysis in children: a pilot project
Background: We describe results of a pilot project to develop a ‘Paediatric Haemodialysis Trigger Tool’ (pHTT) for children on chronic intermittent haemodialysis to enable regular monitoring of adverse events.
DESIGN & OUTCOMES:
 Single-centre
	 quality improvement
 project
Two cycles of 8 weeks each
15 children in Cycle 1 and 13 in Cycle 2
Median age 8.7 years
182 individual haemodialysis
sessions reviewed
Reduction in harm rate per session from 1.03 to 0.32 [P < 0.001]
Balasubramanian et al. 2022
CONCLUSION: This pilot feasibility and utility study demonstrates possible benefits of a straightforward and potentially useful bedside tool to monitor adverse events during haemodialysis in children.
